# Caloric restriction mimetic 2-deoxyglucose alters metabolic and transcriptomic phenotype in association with changes in chromatin accessibility in human astrocytes

**DOI:** 10.1038/s41598-025-03796-w

**Published:** 2025-06-03

**Authors:** Matthew Spencer, Jacqueline R. Kulbe, Vikram Venkatesh, Anna Laird, Mary Ford, Sydney O’Brien, Ali Boustani, Johannes C. M. Schlachetzki, Jerel Adam Fields

**Affiliations:** 1 Department of Psychiatry, University of California, San Diego, CA USA; 2 Department of Neurosciences, University of California, San Diego, CA USA

**Keywords:** Astrocytes, Immunometabolism, Chromatin accessibility, Glycolysis, Caloric restriction, Neuroinflammation, Astrocyte, Transcriptomics

## Abstract

Caloric restriction and ketogenic diets may modify the progression of neurological disorders, including HIV-associated neurological disorders and Alzheimer’s disease, in part by influencing astrocyte function. This study examines how metabolic substrate availability affects metabolic processes and gene expression in human astrocytes. We exposed astrocytes to the glycolysis inhibitor 2-deoxyglucose (2-DG), to mimic caloric restriction, prior to stimulation with interleukin-1β and measured extracellular flux using the Seahorse ® platform. We next analyzed gene expression and chromatin accessibility changes using RNA-sequencing and ATAC-sequencing, respectively. Finally, we tested the effects of glucose deprivation and the ketone body β-hydroxybutyrate (BHB) on inflammatory gene expression. 2-DG reduced oxygen consumption rate and extracellular acidification rate in the presence of IL-1β, while concomitantly decreasing expression of pro-inflammatory cytokines TNF, IL-6, and C3. These changes were linked to altered chromatin structure. The metabolic substrate β-hydroxybutyrate was associated with reduced cytokine expression compared to glucose. Inhibition of glycolysis attenuated IL-1β-induced inflammation and gene expression changes and altered chromatin architecture. Both glucose deprivation and BHB treatment reduced inflammatory cytokine expression, with additive effects when combined with 2-DG. These results suggest that targeting glycolysis could provide therapeutic strategies for treating neurological diseases through modulation of astrocyte-driven inflammation.

## Introduction

Over three billion people worldwide suffer from neurological disorders^1^. Inflammatory processes and metabolic dysfunction are commonly implicated in the pathogenesis of acute and chronic neurological diseases. Astrocytes, the most abundant glial cells in the central nervous system, have been linked to playing important roles in brain homeostasis and disease. They are crucial for maintaining brain health through their numerous essential functions, including providing metabolic support to neurons^2^. Astrocytes communicate with neurons in the brain, for example, by generating lactate to support synaptic activity^3^. Astrocytes actively participate in immune responses within the brain, responding to various physiological and pathological stimuli^4^. Uncontrolled or prolonged inflammation in astrocytes can lead to detrimental consequences, contributing to the pathogenesis of neuroinflammatory disorders^5,6^, chronic neurodegenerative diseases, such as Alzheimer and Parkinson diseases, and acute neurological diseases like ischemic injury and traumatic brain injury^7–10^. One underlying mechanism of how astrocytes impact neuronal function in the context of these diseases is a heightened inflammatory response. An increased astrocytic inflammatory response has been associated with altered catabolic reactions^11,12^. Specifically, the metabolic milieu surrounding astrocytes has been implicated in modulating their reactivity and subsequent contribution to inflammatory cascades^13,14^. Caloric restriction (CR) and ketogenic diets (KD) have garnered scientific interest for their potential anti-inflammatory effects^15–17^. CR, defined by a reduction in caloric intake without malnutrition, is known to modulate metabolic pathways and enhance cellular stress resistance^16^. A recent study showed that time-restricted caloric intake reduced plaque load and inflammation in an amyloid mouse model of Alzheimer disease^18^. Recently it was shown that glycolysis in astrocytes was suppressed in response to oligomeric amyloid and tau. Enhancing astrocytic glycolysis by targeting IDO1, rate-limiting enzyme in the kynurenine pathway improved metabolic support of astrocytes on neurons and reduced the severity of amyloid and tau pathologies^19^. We recently demonstrated that the CR mimetic and glycolysis inhibitor, 2-deoxyglucose (2-DG), attenuated interleukin-1β (IL-1β)-induced upregulation of pro-inflammatory cytokine gene expression, including IL-1β, IL-6, and tumor necrosis factor-α (TNF-α)^15^, in astrocytes. Similarly, KD, a low-carbohydrate, high-fat diet that induces a state of ketosis, has been associated with reduced inflammation in astrocytes^20,21^. β-hydroxybutyrate (BHB), a ketone body elevated during ketosis, appears to exert anti-inflammatory effects by inhibiting the NLRP3 inflammasome and modulating nuclear factor-kappa B (NF-κB) signaling^22,23^. These dietary interventions hold promise as strategies to mitigate neuroinflammation in human astrocytes, offering insights into potential therapeutic avenues for neuroinflammatory disorders. Here, we tested the hypothesis that 2-DG will inhibit glycolysis (i.e. reduce extracellular acidification rate (ECAR)) and will attenuate IL-1β-induced inflammatory cytokine gene expression in human astrocytes. Additionally, we hypothesized that IL-1β-induced inflammatory gene expression would be attenuated by the absence of the glycolytic substrate, glucose, or by the presence of the alternative substrate, BHB. To address these questions, we measured oxygen consumption rate (OCR) and extracellular acidification rate (ECAR) using the Seahorse assay. We performed multiomics (RNA-seq + ATAC-seq) to unravel distinct changes in gene expression and chromatin configuration in human astrocytes challenged with IL-1 β and 2-DG.Our findings indicate that IL1B dramatically alters astrocytic metabolism and induces a shift from oxidative phosphorylation to glycolysis. The metabolic changes are associated with altered gene expression and chromatin landscapes in astrocytes. Modulation of glycolysis by 2-DG leads to modest decreases in inflammatory gene expression. These findings not only shed light on the direct impact that metabolic substrates have on astrocytic inflammatory responses but also reveal potential underlying regulatory mechanisms at the transcriptional and chromatin levels.

## Materials and methods

### Generation of human astrocytes

This study was approved by the University of California, San Diego Human Research Protections Program and deemed IRB exempt (Federal-wide Assurance #00,000,021 and Institutional Review Board #IORG0000210 [7 March 2019]). All methods were performed in accordance with relevant guidelines and regulations. All data presented for this study used astrocytes that were from a differentiated cell line originally generated prior to 5 June 2019 (as per NIH NOT-OT-19–128) from fetal human brain tissue from terminated pregnancy between 12 and 16 weeks of gestation, as previously described^24^, Donors gave written informed consent for research use of the cells and tissue.

### Treatment of astrocytes

Astrocytes were cultured in 24-well plates at 120,000 cells/well in DMEM (Corning, cat. no. 10013cv) with 10% fetal bovine serum (FBS) (Thermofisher, cat no. 16000036) and 1% antibiotic–antimycotic (anti-anti) (Thermofisher, cat no. 15240062) prior to treatment. Stock media contains 4.5 g/L glucose (25 mM). To mimic caloric restriction and inhibit glycolysis, astrocytes were treated with varying concentrations of 2-DG (10, 20, and 50 mM) inside of incomplete DMEM for 24 h. Incomplete DMEM refers to a basal formulation of DMEM that lacks key components required for cellular metabolism and survival, such as glucose, amino acids, vitamins, and other essential nutrients. To determine whether different metabolic substrates affected cellular response to inflammation, parallel experiments were performed in which the media was supplemented with either glucose (0.45 g/L/2.5 mM) or BHB (30 mM). To model an inflammatory environment, astrocytes were treated with IL-1β for 24 h at a concentration of 20 ng/mL.

### Extracellular flux analyses

Astrocytes were split into a seahorse plate at 3 × 10^4^ cells/well DMEM with 10% FBS and 1% anti-anti. Astrocytes were treated with IL-1β (20 ng/mL), 2-DG (10 mM – 50 mM) or the combination for 24 h. On the day of the assay, media was replaced by fresh incomplete DMEM containing glucose and pyruvate. Cultures were incubated in a non-CO_2_ incubator at 37 °C to equilibrate for approximately 60 min prior to assay. Baseline measurements of oxygen consumption rate (OCR) and extracellular acidification rate (ECAR) were taken prior to the addition of oligomycin (2 μM) (Sigma-Aldrich, cat no. 75351), followed by two additions of FCCP (250 nM) (Sigma-Aldrich, cat no. C2920). After each addition of FCCP, three readings were taken before the injection of the subsequent addition. Maximum oxygen consumption rate was recorded, and the highest reading was recorded to determine maximal mitochondrial respiration, which was after two consecutive injections of 250 nM FCCP. ECAR was automatically calculated and recorded by the Seahorse XFe96 software. Rates were calculated by the Seahorse analyzer, reported as pM O_2_/minute and log of H^+^ production rate, respectively. Samples were run in biological replicates of four in three independent experiments. Biological replicates refer to independent measurements taken from distinct samples with identical treatments.

### RNA isolation and Taqman® human inflammation array and real-time reverse transcription polymerase chain reaction (RT^2^ PCR)

Following the 24 h treatment of IL-1β (20 ng/mL) on astrocytes cultured in 24-well plates, the medium was removed from the wells and cells were washed once with phosphate-buffered saline (PBS). RNA was extracted using the Direct-zol RNA Miniprep Kit (Zymo Research, Irvine, CA, USA, cat. no. R2051) according to the manufacturer’s instructions. A spectrophotometer was used to analyze the purity and concentration of RNA samples. For the RT^2^PCR, RNA was reverse transcribed into cDNA with a high-capacity cDNA Reverse Transcription Kit (Life Technologies™, cat no. 4368814) per the manufacturer’s instructions. Gene expression was determined using RT^2^PCR TaqMan gene expression assays with the QuantStudio 3 sequence-detections system (Life Technologies™) using Taqman primers specific to IL-6 (cat. no. HS000174131_m1), TNFα (cat. no. Hs00174128_m1), and C3 (cat. no. HS00163811_m1). An ActB (Applied Biosystems™, Waltham, MA, USA, cat. no. 1612290) primer was used as a normalization control. A master mix was created using 2 × fast advanced master mix (Thermo Fisher Scientific, Waltham, MA, USA, cat. no. 4444557), 20 × primers, and water. Each reaction well of a microamp fast optical plate (Applied Biosystems™, cat. no. 4346907) received 8.5 µL of the master mix and 1.5 µL cDNA. The reactions were carried out at 95 ℃ for 20 s, followed by 40 cycles of 95 ℃ for 1 s and 60 ℃ for 20 s. Each sample was analyzed in duplicate and their C_T_ values were collected, exported to an Excel file, and used to calculate fold changes using the comparative C_T_ method^25^.

### RNA-seq

#### *RNA-seq library preparation*

RNA-seq libraries were prepared as previously described^26^. Approximately ~ 200,000 human astrocytes were collected in TRIzol. Total RNA was extracted from homogenates and cells using the Direct-zol RNA MicroPrep Kit (Zymo Research R2052) and stored at −80 °C until RNA-seq library preparation. mRNAs were enriched by incubation with Oligo d(T) Magnetic beads (NEB, S1419S) in 2X DTBB buffer (20 mM Tris–HCl pH 7.5, 1 M LiCl, 2 mM EDTA, 1% lithium dodecyl sulfate, 0.1% Triton X-100) at 65 °C for 2 min and were incubated at room temperature while rotating for 15 min. Beads were washed 1 × with RNA Wash Buffer 1 (10 mM TrisHCl pH 7.5, 0.15 M LiCl, 1 mM EDTA, 0,1% lithium dodecyl sulfate 0.1% Triton X-100) and 1 × with RNA Wash Buffer 3 (10 mM Tris–HCl pH 7.5, 0.15 M NaCl, 1 mM EDTA) before elution in RNA Elution Buffer (10 mM Tris–HCl pH 7.5, 1 mM EDTA) at 80 °C for 2 min. PolyA selection was performed a second time, and samples were washed 1 × with Wash Buffer 1, 1 × with Wash Buffer 3, and 1 × with 1 × SuperScript III first-strand buffer. Beads were resuspended in 10 µL 2 × SuperScript III buffer plus 10 mM DTT, and RNA was fragmented at 94 °C for 9 min and immediately chilled on ice before the next step. For first-strand synthesis, 10 µL of fragmented mRNA, 0.5 µL Random primers (50 µM)(Thermo Fisher), 0.5 µL SUPERase-In (Ambion), 1µL dNTPs (10 mM), and 1µL of DTT (10 mM) were heated at 50 °C for one minute. At the end of incubation, 5.8 µL of water, 1 µL of DTT (100 mM), 0.1 µL Actinomycin D (2 µg/uL), 0.2uL of 1% Tween-20 (Sigma) and 0.5 µL of SuperScript III (Thermo Fisher Scientific) were added and incubated in a PCR machine using the following conditions: 25 °C for 10 min, 50 °C for 50 min, and a 4 °C hold. The product was then purified with RNAClean XP beads (Beckman Coulter) according to manufacturer’s instruction and eluted with 10 µL nuclease-free wate. The RNA/cDNA double-stranded hybrid was then added to 1.5 µL Blue Buffer (Enzymatics), 1.1 µL of dUTP mix (10 mM dATP, dCTP, dGTP and 20 mM dUTP), 0.2 µL RNase H (5U/uL), 1.05 µL of water, 1 µL of DNA Polymerase I (Enzymatics) and 0.15 µL of 1% Tween-20. The mixture was incubated at 16 °C overnight. The following day, the dUTP marked dsDNA was purified using 28 µL of SpeedBeads (GE Healthcare), diluted with 20% PEG8000, 2.5 M NaCl to a final concentration of 13% PEG, and eluted with 40 µL elution buffer (DNA elution buffer from Zymo ChIP Clean and Concentrator Kit). The purified dsDNA underwent end repair by blunting, A-tailing, and adaptor ligation as previously described^27^ using barcoded adapters (NextFlex, Bioo Scientific). Libraries were PCR amplified for 16 cycles, size for 200–500 bp size range, quantified using a Qubit dsDNA HS Assay Kit (Thermo Fisher Scientific) and sequenced on a NovaSeq 6000 for 51 cycles according to the manufacturer’s instructions.

### RNA-seq analysis

FASTQ files from RNA-seq experiments were mapped to hg38 using STAR with default parameters. After mapping, tag directories were created using the HOMER command makeTagDirectory. The gene expression raw counts were quantified by HOMER’s^28^ analyzeRepeats command with the option “-condenseGenes -count exons -noadj”. Differential gene expression was calculated using the HOMER command “getDiffExpression.pl”. TPM (transcript per kilobase million) were quantified for all genes matching accession number to raw counts. Differentially expressed genes were assessed with DESeq2^29^ at p-adj (adjusted p-value) < 0.05 and FC (fold change) > 2 where indicated. Genes with TPM < 4 in all conditions were removed from the analysis. Gene ontology enrichment analyses were performed using Metascape^30^.

### ATAC-seq

Human astrocytes were collected as described above and resuspended in 50 µL of ice-cold lysis buffer (10 mM Tris–HCl, pH 7.4, 10 mM NaCl, 3 mM MgCl_2_, 0.1% IGEPAL CA-630). Cells were then spun down at 500 xg for 10 min at 4 ℃. Supernatant was discarded and a transposition reaction was performed on the cell pellet using the Illumina Tagment DNA enzyme and buffer kit (Illumina, San Diego, CA, USA, cat. no. 20034197). Samples were purified using the Zymo ChIP DNA clean and concentrator kit (Zymo Research, Irvine, CA, USA, cat. no. D5205) and transposed DNA was eluted in an elution buffer. Three replicates were completed for the experiment.

### ATAC-seq analysis

ATAC-seq FASTQ files were trimmed to 30 bp prior to mapping to hg38 genome with Bowtie2 with default parameters^31^ HOMER was used to convert aligned reads into ‘‘tag directories’’ for further analysis^28^ To call peaks, we used HOMER’s findPeaks with “*-style factor -size 200 -minDist 200 – L 0 – C 0 -fdr 0.9”.* Next, we ran IDR pairwise on ATAC-seq for each condition. We applied Irreproducibility Discovery Rate (IDR) analysis for replicates. We used DESeq2 using HOMER’s getDIffExpression to identify differential chromatin accessibility with fold change > 2 and p-adj < 0.05. Motif analysis was performed on differential peaks with more than 16 tags using HOMER’s findMotifGenome.pl^28^. The UCSC genome browser was used to visualize ATAC-seq data.

### Statistical analysis

Statistical analysis was conducted in Prism 10.1.1 or R version 4.3.3. *RT*^*2*^*PCR* results were analyzed by two-way ANOVA to determine the effect of interaction between treatment groups, followed by post-hoc Tukey’s or independent unpaired t-tests. Extracellular flux analyses were analyzed by one-way ANOVA followed by post-hoc Tukey’s. p-values < 0.05 were considered significant.

## Results

### 2-DG decreases IL-1β stimulated oxygen consumption rate (OCR) and extracellular acidification (ECAR)

**Fig. 1:**
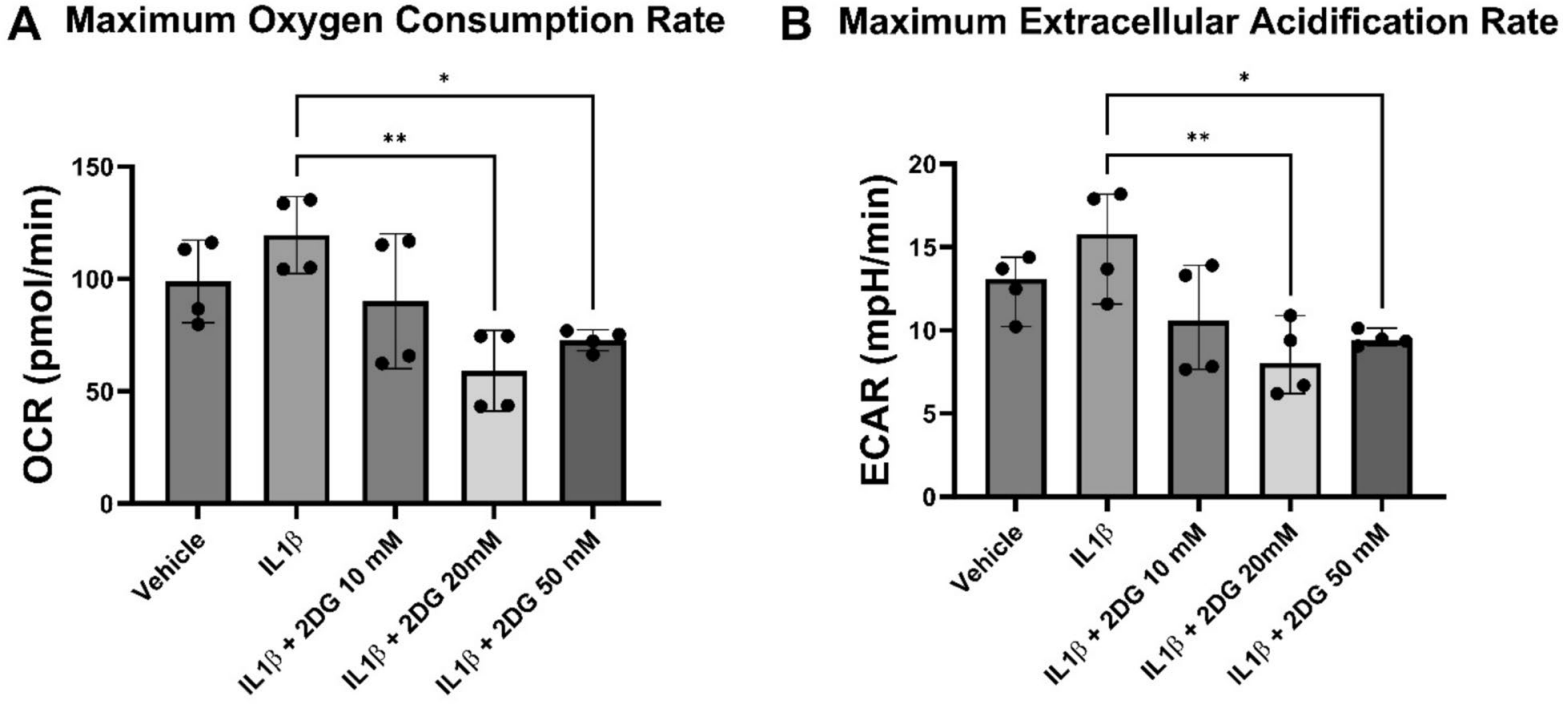
2-DG decreases IL-1β stimulated oxygen consumption rate, a measure of oxidative phosphorylation, and extracellular acidification, a measure of glycolysis. Human astrocytes grown in DMEM were treated with IL-1β (20 ng/mL) +/- 2-DG (10 mM, 20 mM, 50 mM) for 24 h. (**A**) 2-DG decreases IL-1β stimulated OCR (**B**) 2-DG decreases IL-1β stimulated ECAR. One-way ANOVA, followed by post-hoc Tukey’s, revealed a significant difference across groups for both OCR (p < 0.001) and ECAR (p < 0.0001). Independent unpaired t-tests between IL-1β and IL-1β + 2-DG treatments p-values. *p < 0.05; **p < 0.01. Mean +/- SEM. *n* = 3–5/group. 2-DG = 2-deoxyglucose. OCR = oxygen consumption rate. ECAR = extracellular acidification rate.

Unchallenged astrocytes rely on oxidative phosphorylation for their energy needs, whereas reactive astrocytes increasingly use glycolysis^32^. First, we asked whether different concentrations of IL-1β and 2-DG alter the metabolic profile in cultured primary human astrocytes. Astrocytes were grown in stock media with a glucose concentration of 25 mM and treated with IL-1β +/- 2-DG at concentrations of 10 mM, 20 mM, and 50 mM. Extracellular flux analyses were conducted to determine the effect of a range of IL-1β and 2-DG concentrations on OCR and ECAR, proxies for oxidative phosphorylation and glycolysis, respectively. OCR and ECAR were measured at baseline and in the presence of the prototypical immune stimulus, IL-1β (20 ng/mL) (*n* = *3–5/group)* after dual injections of FCCP (250 nM) **(Supplementary Fig. S1)**. FCCP was selectively used as a mitochondrial inhibitor to allow for continued mitochondrial respiration to occur. One-way ANOVA, followed by post-hoc Tukey’s, revealed a significant difference across groups for both OCR (p < 0.001) and ECAR (p < 0.0001). IL-1β non-significantly increased OCR compared to vehicle by 21%. 2-DG did not significantly alter baseline OCR. However, the highest concentration of 2-DG (50 mM) non-significantly decreased OCR by 22% when compared to vehicle. The addition of 20 mM (p < 0.01) or 50 mM (p < 0.05) 2-DG to IL-1β significantly decreased OCR compared to IL-1β alone **(**Fig. 1A**)**. IL-1β non-significantly increased ECAR compared to vehicle by 36%. 2-DG did not significantly alter baseline ECAR. However, the highest concentration of 2-DG used, 50 mM, non-significantly decreased ECAR by 26%. The addition of 20 mM (p < 0.01) 2-DG to IL-1β significantly decreased ECAR compared to IL-1β alone **(**Fig. 1B**)**. Taken together, 2-DG attenuated IL-1β -induced astrocytic metabolic activation.

### 2-DG attenuates the expression of IL-1β induced inflammatory genes and alters metabolic gene expression compared to vehicle

**Fig. 2:**
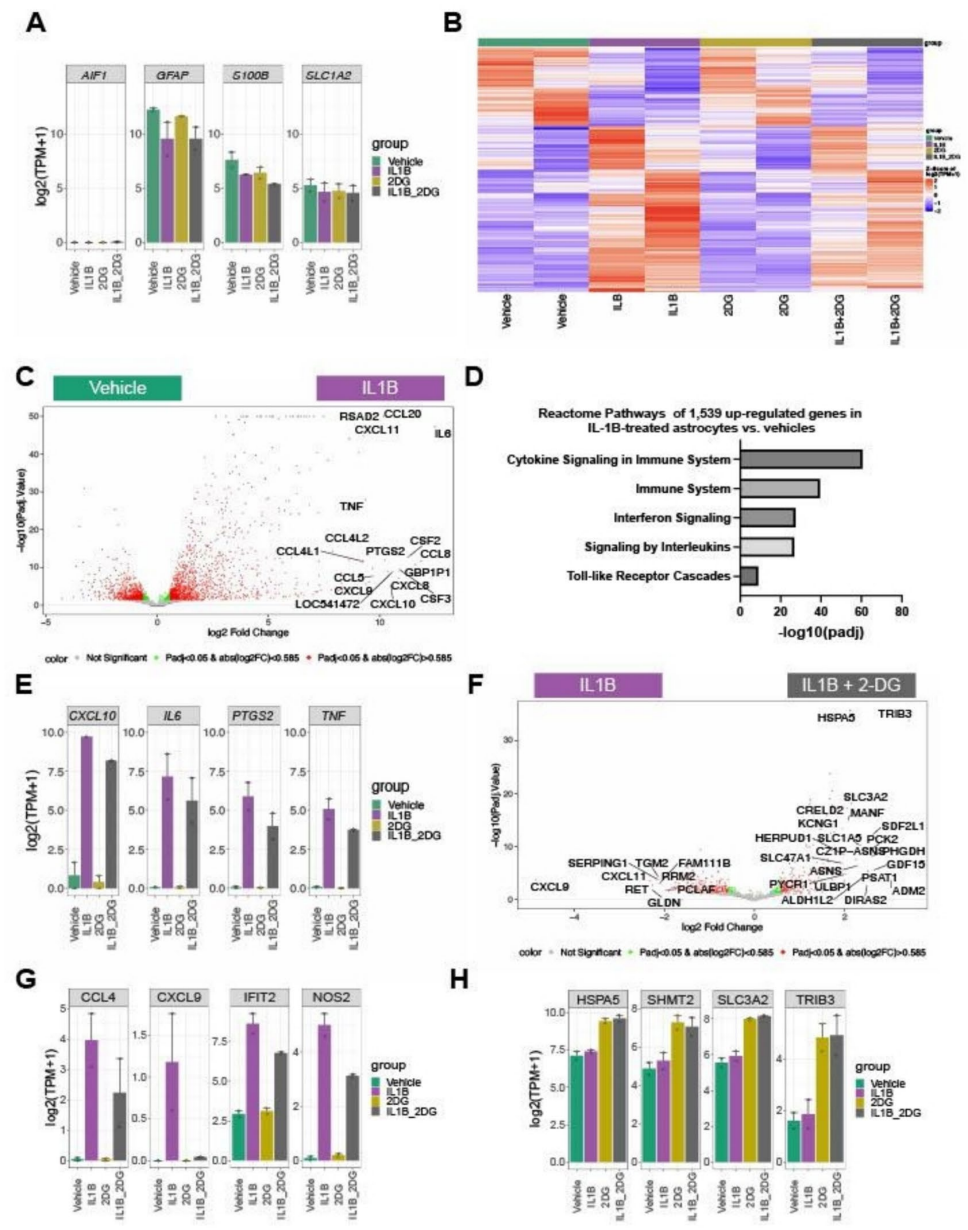
IL-1β induces a pro-inflammatory gene expression profile in cultured human astrocytes. (**A**) Bar graph of expression of the macrophage/microglia and astrocyte marker genes (AIF1, GFAP, S100B, SLC1 A2). (**B**) Heatmap of gene expression changes across vehicle, IL-1β, 2DG, and IL-1β + 2DG treated astrocyte cultures. (**C**) Volcano plot of log2-fold gene expression changes and -log10(FDR) between vehicle and IL-1B treated astrocyte cultures (log2 fold change > 1 &; FDR < 0.05). (**D**) Reactome analysis of differentially up-regulated genes in IL-1β vs. vehicle. (**E**) Bar graphs of expression of inflammatory genes (CXCL10, IL6, PTGS2, TNF). (**F**) Volcano plot of log2-fold gene expression changes and -log10(FDR) between vehicle IL-1β vs IL-1β + 2DG treated astrocyte cultures. (**G**) Bar graphs of examples of genes downregulated by IL-1β + 2-DG compared to IL-1β. (**H**) Bar graphs of examples of genes upregulated by 2-DG and IL-1β + 2-DG compared to vehicle or IL-1β.

To explore the effect of IL-1β and 2-DG on human astrocyte gene expression, human astrocytes were treated with vehicle (incomplete DMEM), IL-1β (20 ng/mL), 2-DG (20 mM), or IL-1β + 2-DG for 24 h and RNA-sequencing and transcriptome analyses were conducted. 20 mM 2-DG was chosen based on our extracellular flux analysis results indicating robust inhibition of glycolysis at this dose and to ensure consistency with previous studies^15^. Human astrocytes showed high gene expression of astrocytic marker genes like *GFAP, S100B,* and *SLC1 A2* (encoding GLT1/EAAT2) (Fig. 2A). Of note, we did not detect any genes such as *AIF1* (encoding IBA1) indicating microglia contamination (Fig. 2A). Next, we performed differential gene expression (DGE) analysis as defined as fold change > 1.5 and adjusted p-value < 0.05 between the four experimental groups (Fig. 2B). We identified 2,495 genes that were differentially expressed between IL-1β-treatment and vehicle. Specifically, 1,539 genes were up-regulated and 956 down-regulated in IL1B-treated astrocytes compared to vehicle (Fig. 2C). Reactome analysis revealed that genes associated with cytokine and interferon signaling were enriched in IL-1β-challenged primary human astrocytes (Fig. 2D). IL-1β treatment of human astrocytes led to a significant increase in the expression of the pro-inflammatory cytokines *TNF*, and *IL6* as well as the chemokine *CXCL10* and the *PTGS2*, a gene involved in prostaglandin synthesis (Fig. 2E).

Next, we determined whether 2-DG impacts gene expression of human astrocytes. 2-DG led to a mild reduction in gene expression reduction of the pro-inflammatory cytokines such as *TNF*, *IL6,* as well as *CXL10* and *PTGS2* IL-1β (Fig. 2E). 2-DG significantly reduced the gene expression of 214 genes in IL-1β-treated astrocytes (Fig. 2F). Genes down-regulated by 2-DG included the chemokines *CCL4* and *CXCL9* (Fig. 2G). Interferon-stimulated genes such as *IFIT2* and genes involved in the production of nitric oxide, e.g., *NOS2,* were significantly down-regulated (Fig. 2F). Reactome analysis showed that 2-DG dampened the expression of genes involved in cholesterol biosynthesis, interferon signaling, and cytokine signaling. 2-DG treatment also increased expression of genes involved in stress response, metabolism, and signaling, e.g., *HSPA5*, *SHMT2*, *SLC3 A2*, and *TRIB3* (Fig. 2H). Taken together, our data indicate that IL-1β administration leads to a dramatic induction of pro-inflammatory cytokines in human astrocytes, a response, which can be dampened by targeting glycolysis via 2-DG.

### ATAC seq indicates that 2-DG alters open chromatin and transcription factor binding sites compared to vehicle-treated human astrocytes, and that the addition of 2-DG to IL-1β alters open chromatin and transcription factor binding sites compared to IL-1β alone

**Fig. 3:**
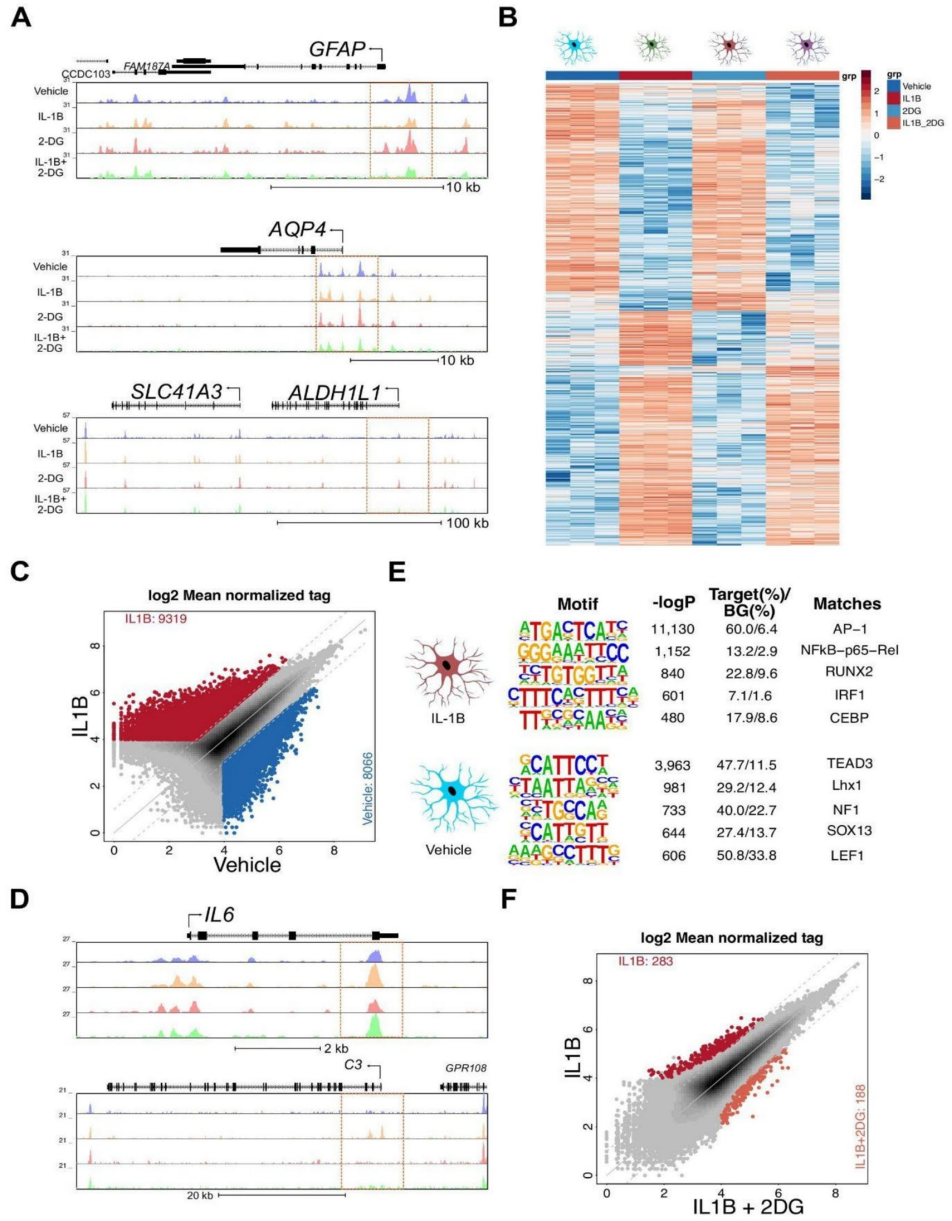
IL-1β alters the chromatin accessibility landscape in cultured human astrocytes. (**A**) UCSC genome browser tracks showing ATAC-seq at the GFAP, AQP4, and ALDH1L1 loci. Extended promoter regions are highlighted. (**B**) Heatmap of changes in chromatin accessibility across vehicle, IL-1B, 2-DG, and IL-1B + 2-DG treated astrocytes. (**C**) Scatter plot of ATAC-seq peaks. Differentially accessible regions (fold change > 2 & adj. p-value < 0.05) are highlighted in red for IL-1β-treated astrocytes and in blue for vehicle-treated astrocytes. (**D**) UCSC genome browser tracks showing ATAC- seq at the IL6 and C3 loci. Differential peaks are highlighted. (**E**) De novo motif analysis of differentially accessible regions in IL-1B-treated astrocytes (top) compared to vehicle- treated astrocytes (bottom). (**F**) Scatter plot of ATAC-seq peaks. Differentially accessible regions (fold change > 2 &; adj. p-value < 0.05) are highlighted in red for IL-1β-treated astrocytes and in orange for IL-1B + 2-DG treated astrocytes.

2-DG partially suppressed the inflammatory response of astrocytes to IL-1β, and we wondered if changes in gene expression were associated with altered chromatin accessibility. To answer this question, we performed ATAC-seq of vehicle, IL-1β, 2-DG, and IL-1β + 2-DG-challenged primary human astrocytes. As expected, chromatin was highly accessible at the promoter loci of astrocyte marker genes such as GFAP, AQP4, and ALDH1L1 (Fig. 3A). Next, we determined differentially accessible intergenic regions as defined by > 3,000 bp from transcription start site (TSS), fold change > 2 and false discovery rate (FDR) < 0.05 (Fig. 3B). We detected ~ 17,000 ATAC peaks that were different between IL-1β-challenged astrocytes and vehicle controls (Fig. 3C). Examples of differentially accessible regions at the *IL6* and *C3* loci are provided in Fig. 3D. Motif analysis of ATAC peaks that are gained in IL-1β revealed enrichment for DNA binding motifs for the AP-1, NfkB and IRF transcription factor families (Fig. 3E, upper panel). ATAC-peaks lost in IL-1β showed transcription factor families important for astrocyte identity such as TEAD, LHX, and SOX (Fig. 3E, lower panel). Similar to the RNA-seq analysis, the changes in chromatin accessibility between IL-1β and IL-1β + 2-DG-challenged astrocytes were mild with only ~ 490 peaks that met the criteria for being differentially accessible (Fig. 3F). Taken together, our data indicates that IL-1β induces a dramatic change in the epigenetic landscape of astrocytes and reveals that AP-1 and NFkB drive the phenotypic change. However, 2-DG did not change the gene expression and epigenetic landscapes.

### IL-1β induced increases in TNFa, IL-6 and C3 mRNA expression are higher in the presence of glucose than without glucose and are attenuated in a dose-dependent manner by 2-DG

We have previously demonstrated that 2-DG has an acute impact on pro-inflammatory gene expression in IL-1β-challenged astrocytes after 6 hours^15^. To determine if this effect was due to glycolytic inhibition, human astrocytes were cultured with (+) glucose (0.45 g/L) or without (-) glucose at varying concentrations of 2-DG, while under IL-1β (20 ng/mL) induced astrogliosis (*n* = 3/group), and TNFα, IL-6, and C3 gene expression were analyzed by RT^2^ PCR.

**Fig. 4:**
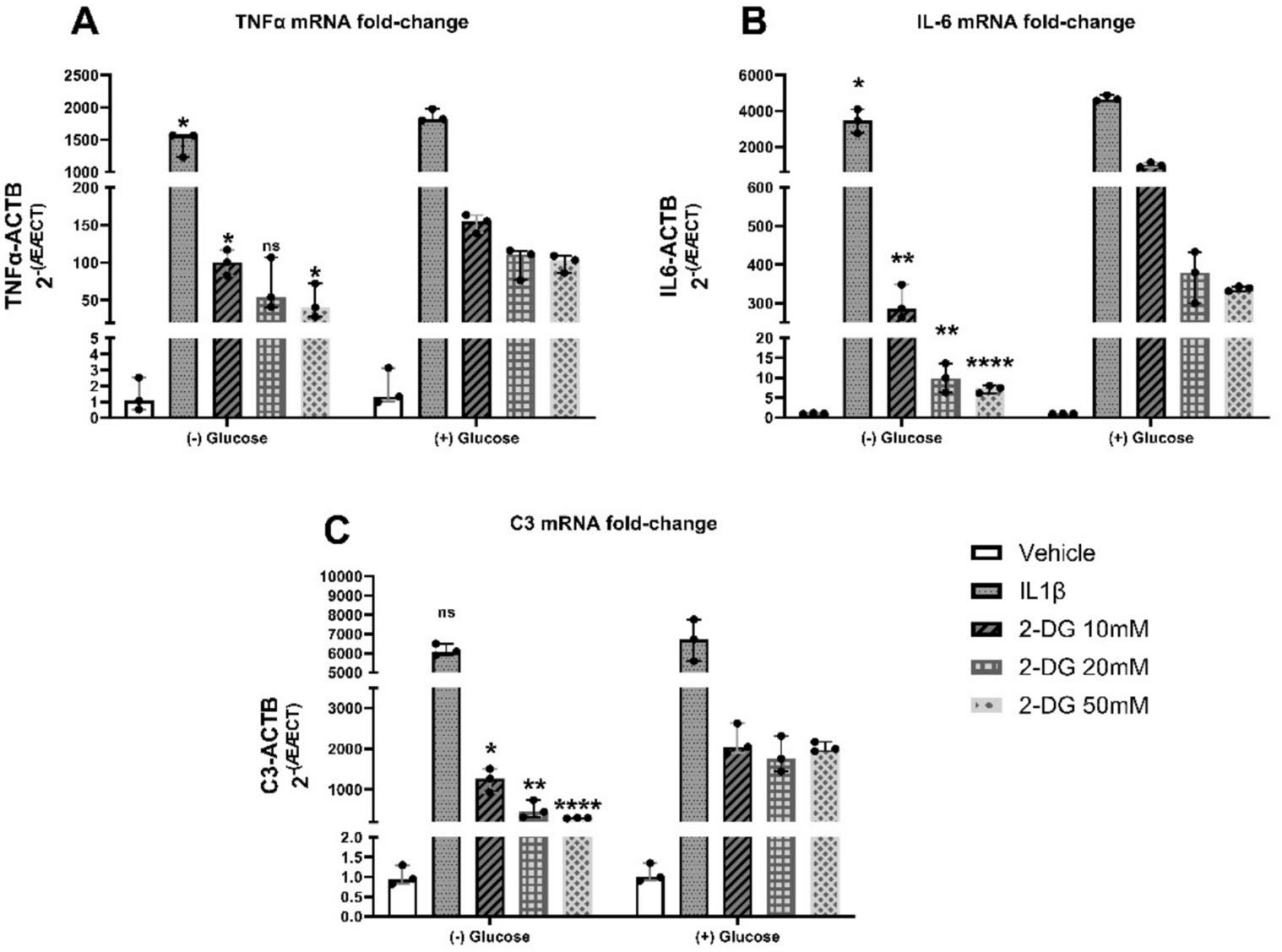
IL-1β-induced increases in TNFa, IL-6 and C3 mRNA expression are higher in the presence of glucose, a glycolytic substrate, than without glucose and are attenuated in a dose-dependent manner by 2-DG. Human astrocytes treated with IL-1β (20 ng/ML) +/- 2-DG (10 mM – 50 mM) for 24 h with glucose (0.45 g/L) and without glucose. Fold change of TNFα (**A**), IL-6 (**B**), and C3 (**C**) mRNA transcript levels normalized to ACTB mRNA levels. Two-way ANOVA (TNFα, p = 0.0003; IL-6, p = 0.0018; C3, p = 0.0161) showed a significant interaction between glucose presence and 2-DG dosage. Independent unpaired t-tests between (+) glucose and (-) glucose treatment p-values. *p < 0.05; **p < 0.01; ***p < 0.0001; n.s. no significance. Median +/- 95% CI. *n* = 3. 2-DG = 2-deoxyglucose.

While under IL-1β (20 ng/mL) induced astrogliosis, 2-DG caused a decrease in inflammatory gene expression across both (+) glucose and (-) glucose treatment groups in a dose-dependent manner **(**Fig. 4**).** Two-way ANOVA showed a significant interaction between glucose presence and 2-DG dosage (TNFα, p = 0.0003; IL-6, p = 0.0018; C3, p = 0.0161). Next, independent unpaired t-tests were conducted to determine the significance between (+) glucose and (-) glucose treatment groups. Overall, cytokine expression was significantly higher in the presence of glucose. For TNFα, IL-1β (p < 0.05), 2-DG 10 mM + IL-1β (p < 0.05), and 2-DG 50 mM + IL-1β (p < 0.05) were significantly different between (+) glucose and (-) glucose treatment groups **(**Fig. 4A**)**. For IL-6, IL-1β (p < 0.05), 2-DG 10 mM + IL-1β (p < 0.01), 2-DG 20 mM + IL-1β (p < 0.01) and 2-DG 50 mM + IL-1β (p < 0.0001) were significantly different between (+) glucose and (-) glucose treatment groups **(**Fig. 4B**)**. For C3, 2-DG 10 mM + IL-1β (p < 0.05), 2-DG 20 mM + IL-1β (p < 0.01) and 2-DG 50 mM + IL-1β (p < 0.0001) were significantly different between (+) glucose and (-) glucose treatment groups **(**Fig. 4C**)**.

### IL-1β induced increases in TNFa, IL-6 and C3 mRNA expression are higher in the presence of glucose compared to β-hydroxybutyrate or no exogenous energy substrate and are attenuated by 2-DG

**Fig. 5:**
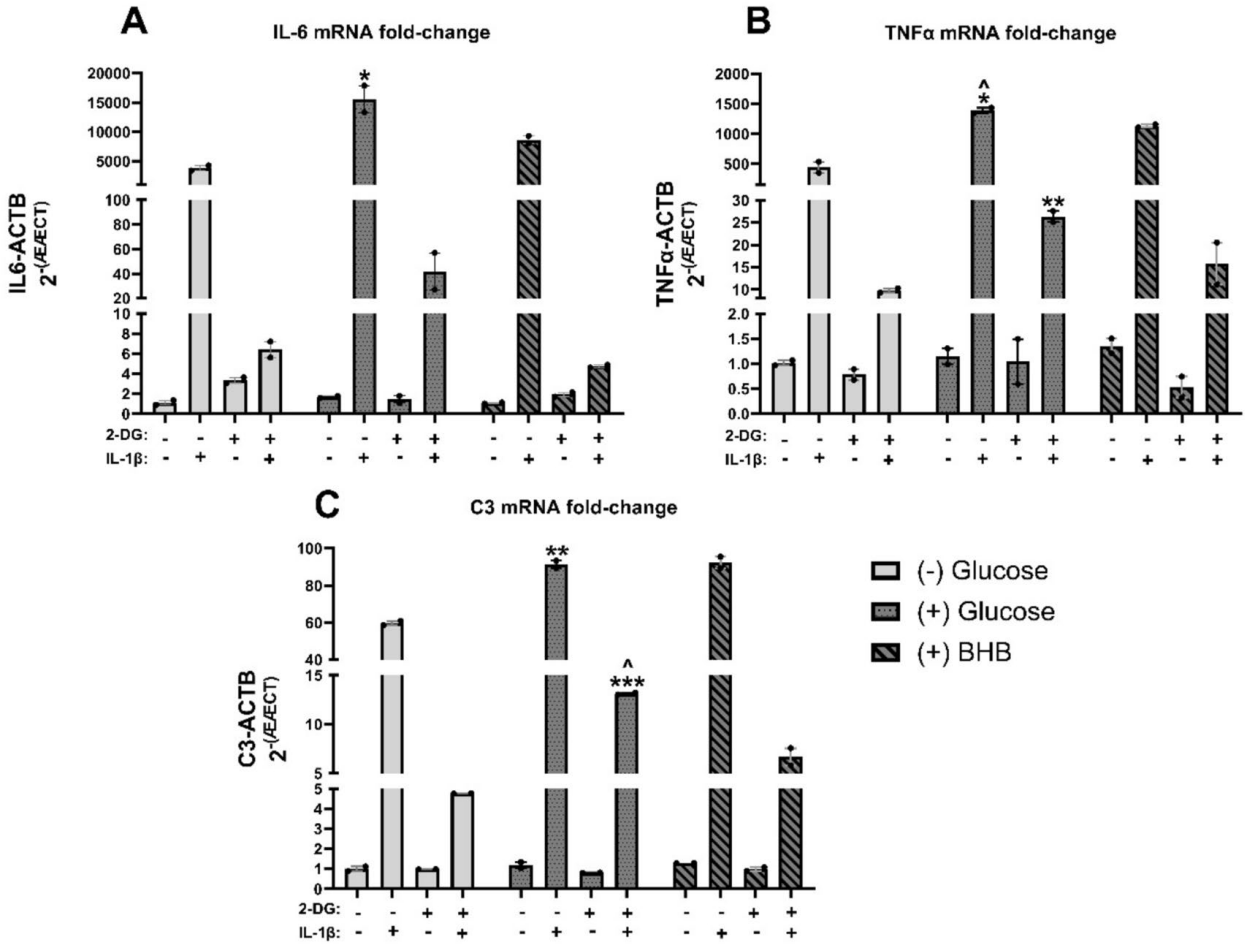
IL-1β induced increases in TNFa, IL-6 and C3 mRNA expression is higher in astrocytes in the presence of glucose compared to β-hydroxybutyrate or no exogenous energy substrate and are attenuated by 2-DG. Human astrocytes treated with IL-1β (20 ng/mL) +/- 2-DG (20 mM) for 24 h with glucose (0.45 g/L), without glucose, or in the presence of BHB (30 mM). Fold Change of (**A**) IL-6, (**B**) TNFα, and (**C**) C3 mRNA transcript levels normalized to ACTB mRNA. Two-way ANOVA revealed a significant interaction between metabolic substrate and 2-DG for IL-6 (p < 0.0001), TNFα (p < 0.0001), and C3 (p < 0.0001). *p < 0.05; **p < 0.01; ****p < 0.0001 between (+) glucose and (-) glucose treatment and ^p < 0.05 between (+) glucose and (+) BHB by independent unpaired t-tests with p-values. Independent unpaired t-tests between (+) glucose and (+) BHB treatment corrected p-values. ^*p* < *0.05*. Median +/- 95% CI. *n* = 2. 2-DG = 2-deoxyglucose. BHB = β-hydroxybutyrate.

To examine the effect of alternative metabolic substrates on cytokine gene expression, human astrocytes were cultured with (+) glucose (0.45 g/L), without (-) glucose, or in the presence of β-hydroxybutyrate (30 mM), a non-glycolytic substrate, and then treated with 2-DG (20 mM) and/or IL-1β (20 ng/mL) (*n* = 3/group). TNFα, IL-6, and C3 gene expression were analyzed by RT^2^ PCR. Overall, while under IL-1β (20 ng/mL) induced astrogliosis, mRNA cytokine expression was higher in the presence of glucose than in the presence of no glucose or β-hydroxybutyrate, and IL-1β induced increases in mRNA cytokine expression were attenuated by 2-DG in all groups **(**Fig. 5**)**. Two-way ANOVA showed a significant interaction between metabolic substrate and 2-DG dosage for TNFα (p < 0.0001), IL-6 (p < 0.0001), and C3 (p < 0.0001). Independent unpaired t-tests between groups were conducted to determine the significance between (+) glucose and (-) glucose treatment groups and (+) glucose and (+) BHB treatment groups. For IL-6, IL-1β (p < 0.05) was significantly different between (+) glucose and (-) glucose **(**Fig. 5A**)**. For TNFα, IL-1β (p < 0.05) and IL-1β + 2-DG (p < 0.01) were significantly different between (+) glucose and (-) glucose, and IL-1β (p < 0.05) was significantly different between (+) glucose and (+) BHB **(**Fig. 5B**)**. For C3, IL-1β (p < 0.01) and IL-1β + 2-DG (p < 0.001) were significantly different between (+) glucose and (-) glucose, and IL-1β + 2-DG (p < 0.05) was significantly different between (+) glucose and (+) BHB **(**Fig. 5C**)**.

## Discussion

Neuroinflammation and metabolic dysfunction contribute to a variety of neurologic disorders including neurodegenerative diseases such as Alzheimer’s disease. Due to their dual role as inflammatory and supportive cells, astrocytes have both neurotoxic and neuroprotective phenotypes^33,34^. Although primarily glycolytic, astrocytes metabolically switch between glycolytic and oxidative metabolism based on external stimuli and metabolic milieu^35^ with each metabolic pathway likely differentially regulating pro-inflammatory and pro-supportive phenotypes^36^. Therapeutic diets, such as CR and KD, have anti-inflammatory effects^15–17^, possibly through modulation of metabolic pathways^16,37,38^. In-depth elucidation of these mechanisms will further the development of neuroprotective interventions that target these pathways.

We previously demonstrated that the glycolytic inhibitor and CR mimetic, 2-DG, attenuates IL-1β-induced upregulation of proinflammatory cytokine gene expression^15^. In this expanded follow-up study, our overall results suggest that in cultured human astrocytes, 24 h immune stimulus with IL-1β induces metabolic activation, alters gene expression and chromatin accessibility, and increases expression of pro-inflammatory cytokines. Effects that are modulated by inhibition of glycolysis with 2-DG, removal of the glycolytic substrate glucose, or treatment with the alternative metabolic substrate BHB. This data suggests that in human astrocytes, glycolysis facilitates inflammatory gene expression, whereas inhibition of glycolysis reduces inflammatory gene expression.

More specifically, our results indicate that treatment with the immune stimulus, IL-1β, non-significantly increases both glycolysis (i.e. ECAR) and oxidative phosphorylation (i.e. OCR), effects that are significantly attenuated at specific dosages by the glycolytic inhibitor, 2-DG, with more robust attenuation of ECAR **(**Fig. 1**)**. Of note, 20 mM 2-DG, the dose chosen for subsequent experiments, did not significantly affect baseline oxidative phosphorylation or glycolysis, is not cytotoxic and can actually enhance astrocyte viability^15^. As hypothesized, inhibition of glycolysis reduced IL-1β-induced inflammatory gene expression **(**Fig. 4, 5**)**. Inflammation induces morphologic changes in astrocytes and increases cytokine production, both energy demanding processes. In fact, in cultured human astrocytes, IL-1β stimulation leads to increased inflammatory gene and protein expression, resulting in concomitant ATP depletion, despite increases in glycolysis and oxidative phosphorylation^39^. Previous studies have demonstrated that inflammatory stimuli, aging, and cellular stress induce astrocytes to metabolically shift from a glycolytic to an oxidative phenotype, allowing for more efficient ATP production^39–42^. Mechanistically, acute increases in energy demand are anticipated to initially be met with increases in glycolysis, a process that is one-hundred times faster than oxidative phosphorylation. However, inhibition of glycolysis forces the cell to rely on alternative metabolic processes, such as ketosis, the process of oxidizing fatty acids to ketone bodies (ex: BHB) to provide alternate substrates for the Krebs cycle (ex: Acetyl CoA)^43,44^, a metabolic process which astrocytes are capable^13^.

CR and KD induce ketosis and have been demonstrated to dampen inflammatory responses, strengthening the possibility that modulation of glycolysis can be used as a therapeutic approach toward inflammation^16,17,45^. There are a number of mechanisms purported to contribute to the neuroprotective effects of CR and KD, including inhibition of the NLRP3 inflammasome, inhibition of histone deacetylases (HDACS), stimulation of endogenous antioxidant systems, improvement of mitochondrial function^17^, and restoration of the function of astrocytes as a neuronal support cell^13^. However, while these mechanisms aid in minimizing hyperinflammation of late-stage disease progression, it is also of note that glucose hypometabolism has been linked to promoted prodromal progression in certain neurodegenerative diseases^46^. This implies that CR and KD diets are likely to have a diverse impact on neuronal health depending on specific disease stages. Due to this, a broader and more comprehensive understanding of the astrocytic pathways impacted by inhibition of glycolysis during immune stimulus is needed.

Therefore, RNA-seq was conducted to assess differential gene expression in an unbiased manner. Our results indicate that cultured human astrocytes differentially expressed over 2,000 genes across vehicle, IL-1β, 2-DG, and IL-1β + 2-DG treatment groups **(**Fig. 2**)** and confirmed our previous data that IL-1β-induced upregulation of the inflammatory genes IL-6 and TNF, and chemokines such as CCL4 was attenuated by 2-DG^15^, suggesting a role for glycolysis in upregulating these processes, an interaction supported by the literature^12,15,16,47,48^. Overall, IL-1β-induced gene pathways included cytokine and interferon signaling. Transcriptomic analysis showed that treatment with 2-DG led to various changes in genes associated with metabolism and stress response, for example *HSPA5/GRP78* (heat shock protein 70 member 5), PDIA6 (protein disulfide isomerase family A member 6), *SHMT2* (serine hydroxymethyltransferase 2), *SLC3 A2* (solute carrier family 3 member 2), and *TRIB3* (tribbles homolog 3) compared to IL-1β or vehicle**.** HSPA5/GRP78 is glucose-regulated^49^ and is induced by caloric restriction^50,51^. Additionally, HSPA5/GRP78 maintains mitochondrial function^52^, reduces LPS-stimulated cytokine production^53^ and enhances AB(1–42) clearance^54^, making it a potential therapeutic target in neurodegenerative disorders^55,56^. SHMT2, a mitochondrial specific isoform, catalyzes the conversion of serine to glycine through one-carbon metabolism. SHMT2 plays a role in DNA methylation and epigenetics^57^, enhances cell proliferation during glucose restriction^58^, is required for mitochondrial respiration, and translation and maintenance of mitochondrially encoded proteins^58^, including complex I^58^. Knock-down of SHMT2 increases inflammatory cytokine production, including IL-6 and TNFa^59^, and dysregulation of one-carbon metabolism has been implicated in the development of neurodegenerative disorders^60^, making SHMT2 a potential therapeutic target for Alzheimer disease^61^.

SLC3 A2, a component of heterometric amino acid transporters, responds to metabolic stress through regulation of amino acid transport^62^, a key function for astrocyte metabolism as astrocytes utilize amino acids anabolically for gluconeogenesis^63^ and catabolically to generate Kreb cycle substrates^64^.

TRIB3, a pseudo kinase, is induced by metabolic stress, including glucose insufficiency^65,66^. TRIB3 is both induced by and a negative regulator of NFkB^67,68^, a key transcription factor of the proinflammatory response^47^. Peripherally, TRIB3 is involved in glucose homeostasis^69^, and in cancer it is known to inhibit fatty acid synthesis in order to promote oxidative lipid decomposition^70^.

Reassuringly, these genes are primarily associated with cellular homeostasis including protein folding, metabolism, amino acid transport, and cell survival^71–77^. They are induced by metabolic, endoplasmic, oxidative, and mitochondrial stress^74,78,79^, pathologic mechanisms known to contribute to the development of neurodegenerative disease^80–82^ and are often upregulated in anaerobic environments requiring sustained energy production such as cancer^74,78,83,84^.

Taken together, the data begins to elucidate the potential mechanistic underpinnings of glycolysis in astrocyte cytokine production and inflammatory response. This insight will enable more refined follow-up studies targeting the identified genes. Many of the aforementioned genes are already known to be induced by metabolic stress and to play important roles in mitochondrial, glucose, amino acid, and fatty acid homeostasis. However, many of the previous studies involving these genes were done in peripheral cell types or cancer cells with limiting data involving astrocytes^70,74,78,83,84^. Therefore, future studies focusing on the role of these genes in immunometabolism and neuroprotection are warranted.

We examined how the inflammatory cytokine IL-1β and the metabolic inhibitor 2-DG affect the chromatin accessibility of human astrocytes. IL-1β was found to dramatically alter the chromatin landscape, increasing accessibility at regions associated with inflammatory genes like IL-6 and C3. This change was likely driven by transcription factors AP-1 and NF-κB. However, while 2-DG partially suppressed the inflammatory response, it had a minimal impact on the chromatin accessibility profile of astrocytes. This suggests that while 2-DG may modulate the inflammatory response, it does not significantly alter the underlying epigenetic mechanisms. Collectively, IL-1β shifted the gene regulatory program of astrocytes into a pro-inflammatory state.

2-DG is a non-metabolizable glucose analogue that competitively inhibits glycolysis^85^, forcing the cell to utilize alternative metabolic substrates, such as fatty acids, in order for the Krebs cycle and oxidative phosphorylation to occur^43,44,85^. However, 2-DG also interferes with the pentose phosphate pathway, impairs glycosylation, and can alter carbohydrate storage^86^. Therefore, 2-DG attenuation of IL-1β- induced upregulation of TNF, IL-1β, and C3 **(**Fig. 2E-G**)** was also assessed under alternative conditions which inhibit or bypass glycolysis, namely, glucose deprivation or administration of the alternative substrate BHB. BHB is a ketone body produced during periods of fasting, low carbohydrate intake, or prolonged exercise, which undergoes specific metabolic pathways distinct from glycolysis to generate substrates for entry into the Krebs cycle and oxidative phosphorylation^87,88^.

Our results found an interaction between 2-DG and glucose presence on inflammatory cytokine mRNA expression **(**Fig. 4**)** with 2-DG decreasing cytokine expression in a dose-dependent manner with or without the presence of glucose. Expectedly, cytokine expression levels are also significantly lower in the glucose restricted groups. Similarly, our results indicate an interaction between 2-DG and metabolic substrate **(**Fig. 5**)**. Expectedly, higher cytokine expression is seen in the presence of glucose compared to BHB, with 2-DG being able to attenuate IL-1β-induced cytokine upregulation across all metabolic substrates (glucose deprivation, glucose, BHB).

With astrocytes playing such an important neuroprotective role^4,7,89,90^, the identification of anti-neuroinflammatory mechanisms is an extremely important point of research regarding numerous neurodegenerative diseases. Overall, these results strengthen the argument for glycolysis being important in the inflammatory response, and that glycolysis represents a crucial therapeutic target for neurodegenerative disorders. Further, these results demonstrate that pharmacologic inhibition of glycolysis can enhance the anti-inflammatory effects of nutrient restriction and suggests that 2-DG or compounds with similar mechanisms of action should be developed and could possibly be used concomitantly to enhance the anti-inflammatory and neuroprotective effects of therapeutic diets such as CR and KD.

While this study offers a comprehensive investigation into the effects of metabolic reprogramming by blocking glycolysis with 2DG in astrocytes on RNA-expression, we currently lack extensive data at the protein level to affirm our findings. Confirming these transcriptional changes at the protein level is an important next step. In addition, it remains unclear how these metabolic changes in astrocytes affect neurons, an important element given the neuroprotective role of astrocytes in the CNS. To further elucidate the effects of metabolic reprogramming throughout the CNS, these experiments should include neuronal, microglial and mixed-culture models. These results could provide a more comprehensive insight into the broader picture of anti-inflammatory effects of CR and KD within the CNS. Interestingly, despite the strong inhibitory effect of 2DG on inflammatory gene expression, glucose deprivation alone did not greatly reduce IL6, TNFa, or C3 expression. One possible explanation is that endogenous glycogen stores sustained glycolytic activity in the absence of external glucose. Future studies should assess glycogen levels in cultured human astrocytes to further explore this possibility.

Another key limitation in this study is that RNA-seq and ATAC-seq were only conducted with 2-DG and that RT^2^PCR experiments included BHB as the only alternative metabolic substrate to glucose. Future experimentation is required with different metabolic substrates, such as fructose, lactate, and acetate, all of which undergo altered decomposition compared to glucose and play an important role in ATP production within astrocytes and neurons. Investigation into specific glucose uptake proteins such as glucose transporter 1 could also illuminate the role glucose has on inflammatory responses separate from its decomposition in glycolysis.

The extracellular flux analyses were conducted in the presence of stock media which contains a glucose concentration of 25 mM. This exceeds euglycemic conditions in the CNS which could influence the OCR and ECAR of the control group. In addition, the use of extracellular flux analyses within this study can also be expanded upon, with the addition of groups treated without glucose and/or with alternative substrates. Examination into the OCR for these alternative substrates would provide more insight into the effects these metabolic substrates have on ATP production within astrocytes. Alternative metabolic substrates on total energy consumption and production in the CNS are key points to study before CR and KD or other pharmacologic glycolysis modulators can be used as therapeutic strategies for the treatment of neurodegenerative disorders. 

## Conclusions

In summary, we have provided a comprehensive investigation into the underlying effects of metabolic reprogramming on RNA expression and chromatin accessibility within human astrocytes. Further investigation remains to be seen regarding other key metabolic substrates used within the CNS. Our findings provide new information. The findings from this study can provide valuable insights into the metabolic adaptations and functional consequences of altered astrocytic metabolism, offering potential therapeutic strategies for targeting neuroinflammatory diseases and neurological disorders associated with disrupted glial function. The integration of RNA-seq and ATAC-seq analyses enhances the depth of our understanding, enabling the identification of candidate genes and pathways that may serve as key mediators in the metabolic regulation of neuroinflammation. Further research elucidating the intricate molecular mechanisms underlying the anti-inflammatory effects of CR and KD on astrocytes will contribute to the development of pharmacotherapies and the refinement of dietary approaches for neuroprotection.

## Supplementary Information

Supplementary Information.

## Data Availability

ATAC-seq and RNA-seq data are available under the accession numbers GSE290342 and GSE290343, respectively (Reviewer token: klqfoqqwzdiznen and uxwhqikyltixpad, respectively). All other datasets used and/or analyzed during the current study are available from the corresponding author upon request.

## Abbreviations

ND: Neurological Disorders
CNS: Central Nervous System
CR: Caloric Restriction
KD: Ketogenic Diets
2-DG: 2-Deoxyglucose
IL-1β: Interleukin-1β
TNFα: Tumor Necrosis Factor-α
BHB: Β-Hydroxybutyrate
NF-κB: Nuclear Factor-Kappa B
ANOVA: Analysis of Variance
RNA-seq: RNA Sequencing
ATAC-seq: Assay for Transposase-Accessible Chromatin with high throughput Sequencing
RT-PCR: Reverse Transcription Polymerase Chain Reaction
ECAR: Extracellular Acidification Rate
OCR: Oxygen Consumption Rate
FBS: Fetal Bovine Serum
Anti-Anti: Antibiotic–Antimycotic
SRC: Spare Respiratory Capacity
PBS: Phosphate-Buffered Saline
IDR: Irreproducibility Discovery Rate
HDACS: Histone Deacetylases
HSPA5/GRP78: Heat Shock Protein 70 Member 5
PDIA6: Protein Disulfide Isomerase Family A Member 6
SHMT2: Serine Hydroxymethyltransferase 2
SLC3 A2: Solute Carrier Family 3 Member 2
TRIB3: Tribbles Homolog 3
TTC17: Tetratricopeptide Repeat Domain 17
ER: Endoplasmic Reticulum
UPR: Unfolded Protein Response
IGF1R: Insulin Like Growth Factor 1
2-DG6P: 2-Deoxy-d-Glucose-6-Phosphate
OXPHOS: Oxidative Phosphorylation
ROS: Reactive Oxygen Species
AcAc: Acetoacetate
TCA: Tricarboxylic Acid
