# Supplementary material for: Caloric restriction mimetic 2-deoxyglucose alters metabolic and transcriptomic phenotype in association with changes in chromatin accessibility in human astrocytes
Source: Sci Rep. 2025 Jun 3;15:19368. doi: 10.1038/s41598-025-03796-w (PMC12134135; doi:10.1038/s41598-025-03796-w)
Supplement: Supplementary file 1 — Supplementary Information. [file 41598_2025_3796_MOESM1_ESM.docx]

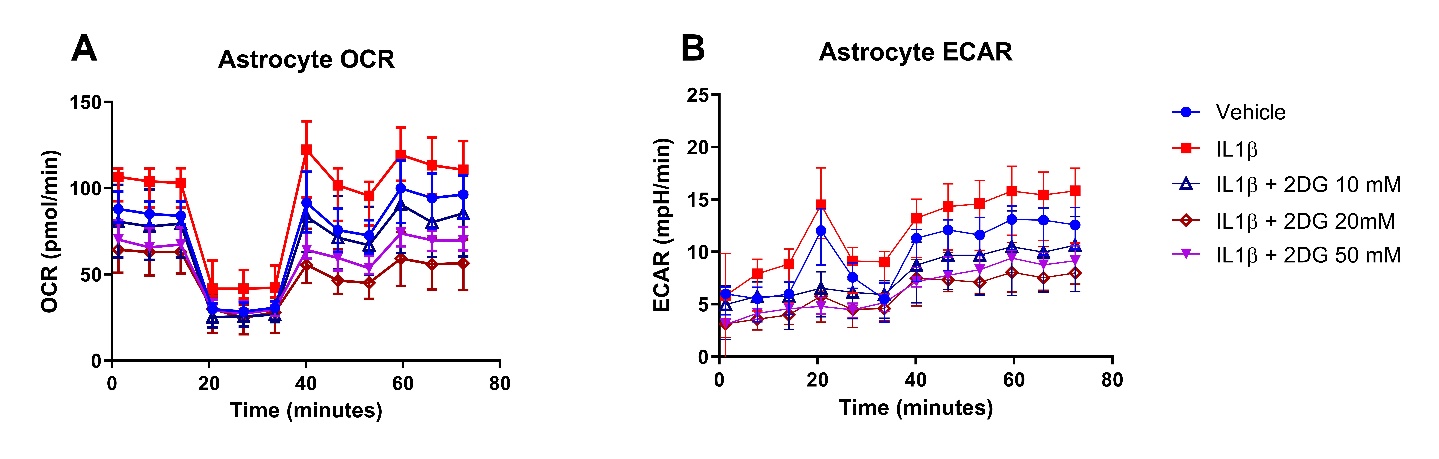


**Supplementary Figure S1.** Human astrocytes grown in DMEM were treated with IL-1β (20 ng/mL) +/- 2-DG (10 mM, 20 mM, 50 mM) for 24h. OCR **(A)** and ECAR **(B)** measurements were taken at baseline, after injection of oligomycin (2 μM), and after two injections of FCCP (250 nM). *n* = 3-5/group. 2-DG = 2-deoxyglucose. OCR = oxygen consumption rate. ECAR = extracellular acidification rate.
